# Tunable Wrinkled Topographies Reveal Feature-Size-Dependent Regulation of Neuronal and Glial Spatial Organization

**DOI:** 10.1007/s12195-026-00953-7

**Published:** 2026-10-25

**Authors:** Owen Gillen, Jouha Min

**Affiliations:** 1 Department of Chemical Engineering, University of Michigan, Ann Arbor, MI, USA; 2 Department of Biomedical Engineering, University of Michigan, Ann Arbor, MI, USA; 3 Macromolecular Science and Engineering Program, University of Michigan, Ann Arbor, MI, USA; 4 Rogel Cancer Center, University of Michigan, Ann Arbor, MI, USA; 5 Biointerfaces Institute, University of Michigan, Ann Arbor, MI, USA; 6 Weil Institute for Critical Care Research and Innovation, Ann Arbor, MI, USA

**Keywords:** Surface topography, Contact guidance, Neurite outgrowth, Schwann cells, MXene, Peripheral nerve regeneration

## Abstract

**Purpose:**

Mechanical cues presented by biomaterial surfaces are powerful regulators of cellular behavior, yet how topographical feature size differentially regulates neuronal and glial spatial organization remains poorly understood. Although anisotropic surface architectures have been shown to promote neural alignment and neurite outgrowth, systematic comparisons across multiple topographical regimes within a common material platform have been limited.

**Methods:**

To address this gap, we developed a scalable wrinkle-based nanofabrication platform capable of generating anisotropic MXene topographies spanning characteristic nano-, submicron-, and micron-scale regimes. By varying multilayer thickness, the platform enabled systematic shifts in characteristic wrinkle dimensions while maintaining a common material composition and surface chemistry.

**Results:**

Using this platform, we found that distinct regenerative processes exhibit different responses across the topographical regimes. While all wrinkled topographies effectively aligned Schwann cells, the nanoscale topographies produced the greatest cellular elongation. In contrast, submicron and microscale topographies showed greater early neuronal alignment, whereas the submicron topography showed the greatest neurite extension while maintaining strong directional guidance following differentiation.

**Conclusions:**

These findings reveal distinct structure–response relationships between topographical regimes and neural guidance behavior. More broadly, this scalable wrinkle-based platform provides a versatile strategy for investigating topography-mediated cell–material interactions and informing the design of next-generation regenerative neural interfaces.

## Introduction

Peripheral nerve injuries (PNIs) affect millions of individuals worldwide and often result in incomplete functional recovery, particularly when regeneration must occur across large nerve defects rather than through direct end-to-end repair [1–3]. Although autografts remain the clinical gold standard for nerve reconstruction [1, 3, 4], their use is limited by donor site morbidity, limited tissue availability, neuroma formation, and incomplete sensory recovery. As a result, substantial effort has focused on developing engineered biomaterials capable of directing and enhancing peripheral nerve repair [3–5].

A critical feature of successful nerve repair is the presence of aligned guidance cues that direct axonal growth toward distal targets. During regeneration, Schwann cells reorganize into highly aligned structures known as Bands of Büngner, which contribute structural and biochemical cues that support regenerating axons [3, 6]. In contrast, many clinically available nerve conduits lack sufficient internal architecture to successfully recreate this anisotropic regenerative environment, contributing to poor outcomes across large defects [4–6]. This has driven an increasing interest in engineering biomaterial interfaces that mimic these aligned guidance structures [7–10].

Among the various design strategies explored, surface topography has emerged as one of the most powerful physical regulators of neural cell behavior. Anisotropic features such as grooves, gratings, aligned fibers, and related one-dimensional architectures have been shown to direct cellular elongation, migration, cytoskeletal organization, and neurite extension through contact-guidance mechanisms [11–19]. Topographical dimensions are also known to influence cellular responses, and classic studies have demonstrated that feature depth can be as important as, or even more important than, feature spacing in determining guidance strength [20–26]. Despite these advances, a fundamental question remains unresolved: what topographical feature sizes best support coordinated axonal and glial regeneration? Successful nerve regeneration requires both aligned Schwann cells and efficient neurite extension, yet the optimal topographical requirements of these regenerative processes may not be identical. A surface architecture that maximizes Schwann cell organization may not necessarily maximize neurite growth or directional guidance. Determining the optimal topographical regime therefore requires systematic evaluation of multiple regenerative cell types across a broad range of topographical dimensions within a common experimental platform. However, most previous studies have focused on a single cell type, a limited range of feature sizes, or distinct materials systems, making it difficult to directly compare neuronal and glial responses across topographical feature sizes. Accordingly, the feature-size-dependent design principles governing Schwann cell organization, neurite extension, and directional guidance remain poorly understood [27].

To address this knowledge gap, we employed a wrinkle-based nanofabrication platform capable of generating controlled anisotropic topographies spanning nano-, submicron-, and micron-scale dimensions (Fig. 1). The system is based on MXene multilayer coatings assembled through layer-by-layer (LbL) deposition and subsequently transformed into wrinkle architectures through thermally induced mechanical buckling [28, 29]. Unlike conventional top-down patterning approaches, this strategy enables systematic modulation of topographical dimensions while maintaining consistent material composition and surface chemistry, thereby enabling systematic investigation of the effects of topographical feature size. Furthermore, the mechanically driven fabrication process is readily scalable and adaptable to substrates with diverse geometries, providing a foundation for future translation to more complex three-dimensional regenerative architectures. MXenes have recently emerged as promising materials for neural biointerfaces because of their favorable physicochemical and mechanical properties [30–34]. Using this platform, we systematically investigated how topographical feature size influences both Schwann cell organization and neuron-like cell behavior using RSC96 Schwann cells and SH-SY5Y cells as established in vitro models [19, 35–48]. We hypothesized that distinct topographical feature sizes would differentially regulate regenerative cellular behaviors and that systematic modulation of wrinkle dimensions would reveal distinct responses across topographical feature-size regimes for Schwann cell organization, neurite extension, and directional guidance. Through quantitative analyses of cellular alignment, elongation, neurite extension, lateral confinement, and trajectory persistence, we sought to identify topographical regimes that optimally support Schwann cell organization and neurite guidance relevant to peripheral nerve regeneration.

## Results

### Fabrication and Characterization of Tunable Wrinkled Topographies

To investigate the effects of topographical feature size on neuronal and glial behavior, we developed a wrinkle-based nanofabrication platform capable of generating anisotropic topographies spanning nano-, submicron-, and micron-scale dimensions. The fabrication strategy combines electrostatic LbL assembly of Ti_3_C_2_T_X_ MXene multilayers with thermally induced mechanical buckling to produce highly aligned wrinkle architectures (Fig. 2a). By varying multilayer thickness, wrinkle dimensions could be tuned while maintaining consistent material composition and surface chemistry.

Multilayer growth was first characterized using UV–vis spectroscopy, with absorbance at 780 nm serving as an indicator of MXene film thickness (Fig. 2b). Absorbance increased nearly linearly with bilayer number, ranging from 0.082 for 1-bilayer coatings to 1.004 for 20-bilayer coatings, confirming reproducible multilayer assembly and controllable film growth. For a fixed film–substrate system, classical buckling theory predicts that wrinkle wavelength increases with film thickness [49]. Consistent with this prediction, SEM revealed well-defined anisotropic wrinkle structures whose wavelength increased with multilayer thickness (Fig. 2c). Mean wrinkle wavelength, defined as the peak-to-peak distance between adjacent wrinkle features, ranged from ~ 500 nm for 1-bilayer coatings to ~ 2500 nm for 20-bilayer coatings (Fig. 2b). The distributions of individual peak-to-peak measurements for the selected Nano, Submicro, and Micro conditions are provided in Fig. S1.

Because increasing bilayer number changes both film thickness and wrinkle dimensions, we next examined the effective mechanical properties of the substrates by measuring apparent DMT modulus using AFM (Fig. S2). The planar MXene LbL-coated PS substrates exhibited apparent moduli comparable to uncoated planar PS, with no clear dependence on bilayer number. Wrinkling of uncoated PS also did not substantially change the measured modulus. In contrast, the wrinkled MXene LbL-coated PS substrates exhibited lower apparent moduli than their corresponding planar counterparts, with an overall trend toward lower apparent moduli as bilayer number and wrinkle dimensions increased.

Based on these structural and mechanical characterizations, four experimental groups were selected for subsequent biological studies: Planar, Nano, Submicro, and Micro, as summarized in Table S1. The Planar condition served as an unwrinkled control, while the wrinkled groups represented distinct characteristic feature-size regimes for comparing neuronal and glial responses.

### Topography-Driven Schwann Cell Alignment

Schwann cells play an important role in peripheral nerve regeneration, forming aligned Bands of Büngner that support regenerating axons across the injury site. We therefore first examined how Schwann cell organization differs across the topographical feature-size regimes by culturing RSC96 cells on the Planar, Nano, Submicro, and Micro substrates and analyzing cellular morphology using IF microscopy and SEM (Fig. 3a). All wrinkled topographies showed pronounced anisotropic cellular organization compared with the Planar control, characterized by elongated morphologies and alignment along the wrinkle axis. Because prominent cracks were visible in some cell-associated SEM images, we also examined the as-prepared substrates to determine whether these features were intrinsic to the topography. The cracks were largely absent from the as-prepared substrates (Fig. S3), indicating that these features were introduced during post-culture SEM processing and were unlikely to have influenced cell–substrate interactions during culture.

Quantitative image analysis confirmed increased cellular elongation on all wrinkled substrates relative to the Planar control (Fig. 3b). Cells cultured on Planar surfaces exhibited a relatively low mean aspect ratio (~2.9), compared with 9.0, 6.3, and 4.8 for the Nano, Submicro, and Micro groups, respectively. The Nano condition therefore showed the greatest degree of elongation. Orientation analysis further demonstrated strong cellular alignment on all wrinkled substrates (Fig. 3c–d). Cells cultured on Planar substrates exhibited broad orientation distributions, whereas ~ 85%, 87%, and 82% of cells on the Nano, Submicro, and Micro substrates, respectively, aligned within 15° of the wrinkle axis.

To further examine whether these morphological differences were associated with focal adhesion organization, vinculin staining was performed. The number and mean area of vinculin puncta showed relatively limited differences across the conditions (Fig. S4). In contrast, puncta orientation differed across the wrinkled substrates, with the Nano condition showing the smallest angular deviation from the wrinkle axis relative to the Submicro and Micro conditions (Fig. 3e). Thus, the Nano condition showed both the greatest cellular elongation and the strongest focal adhesion alignment.

### Topography-Driven Early Neuronal Organization

To examine early neuronal responses before extensive neurite differentiation, SH-SY5Y cells were cultured on Planar, Nano, Submicro, and Micro substrates for 24 h in complete growth medium and analyzed using IF microscopy and SEM (Fig. 4a). At this early time point, all wrinkled topographies showed greater anisotropic cellular organization relative to the Planar control.

Orientation analysis revealed clear differences across the topographical regimes (Fig. 4b–c). Cells cultured on Planar substrates exhibited broad orientation distributions, whereas the Submicro and Micro groups showed the greatest alignment, with 83% and 78% of cells, respectively, aligned within 15° of the wrinkle axis, compared with 48% for the Nano group. Process length also showed modest differences across conditions after 24 h (Fig. 4d). Mean process lengths ranged from 33.5 μm on Planar substrates to ~ 38–42 μm on wrinkled substrates, with the Submicro group showing the greatest mean length (42.0 μm). Although the absolute differences were modest, process length was significantly greater on the Submicro topography than on the Nano and Micro topographies.

Vinculin staining was performed to assess focal adhesion organization. In contrast to the discrete vinculin puncta observed in Schwann cells, SH-SY5Y cells exhibited a more diffuse or plaque-like vinculin distribution (Fig. S5). On the wrinkled substrates, these vinculin-positive adhesion structures appeared preferentially oriented along the wrinkle axis; however, because individual structures could not be reliably segmented, this orientation was not quantitatively analyzed.

### Topography-Driven Neurite Guidance

Successful peripheral nerve regeneration requires regenerating axons to extend over long distances while maintaining directional fidelity toward distal targets. We therefore next examined how neurite guidance differs across the topographical feature-size regimes following neuronal differentiation. SH-SY5Y cells were cultured and differentiated on the different substrates, and neurite organization was quantified using SEM and trajectory analysis to evaluate directional persistence, lateral confinement, and neurite extension.

SEM imaging and neurite trajectory mapping revealed distinct neurite–topography interactions across the experimental groups (Fig. 5a, b and Fig. S6). Neurites on Planar substrates exhibited broad radial trajectories, whereas Nano topographies showed modest alignment with greater lateral exploration. In contrast, Submicro and Micro topographies showed pronounced neurite alignment and more confined trajectories along the wrinkle direction. Similar trends were observed for cell body orientation, with elongated somas oriented parallel to the wrinkle axis on the larger-feature topographies.

Quantitative trajectory analysis supported these observations. Lateral deviation decreased from 18.5 μm on Planar substrates to 12.7, 7.4, and 6.2 μm on the Nano, Submicro, and Micro groups, respectively (Fig. 5c), consistent with greater lateral confinement on the Submicro and Micro topographies. Similarly, neurite persistence ratio increased from 0.86 on both Planar and Nano substrates to 0.93 and 0.92 on the Submicro and Micro groups, respectively (Fig. 5d). Neurite length also differed across the topographical regimes (Fig. 5e), with the Submicro group showing the greatest mean length (112 μm), compared with Planar (58 μm), Nano (64 μm), and Micro (73 μm). Thus, while Submicro and Micro topographies showed comparable directional guidance, the Submicro topography showed the greatest neurite extension.

## Discussion

Peripheral nerve regeneration depends on the coordinated responses of Schwann cells and regenerating axons to both biochemical and physical cues. Although anisotropic surface architectures, including microgrooves, aligned fibers, and patterned substrates, have been shown to promote neural alignment and neurite outgrowth, it remains unclear how topographical feature size differentially influences the multiple cellular processes required for regeneration. Direct comparisons across topographical feature sizes have been challenging because previous studies often employed different fabrication methods, material systems, or surface chemistries. Because each condition contained a distribution of local wrinkle wavelengths with some overlap between groups, the Nano, Submicro, and Micro conditions are best considered characteristic feature-size regimes rather than discrete feature sizes. AFM measurements further showed that wrinkle formation altered the effective local mechanical response, with an overall trend toward lower apparent moduli at larger wrinkle dimensions. Thus, topographical dimensions and local mechanics are coupled in this system and may both contribute to the observed cellular responses. Our results show that different regenerative processes exhibit distinct sensitivities to topographical regimes: the Nano condition showed the greatest Schwann cell elongation, Submicro and Micro conditions showed the strongest early neuronal alignment, and the Submicro condition showed the greatest neurite extension while maintaining strong directional guidance. These findings suggest a structure–response relationship between topographical feature-size regimes and neural guidance behavior.

An important finding of this study is that Schwann cell alignment and elongation did not exhibit identical responses to topographical feature size. While all anisotropic topographies showed strong Schwann cell alignment, the Nano group produced the greatest cellular elongation. Our vinculin analysis provides one possible interpretation for this difference, as focal adhesion alignment was strongest on Nano, whereas adhesion number and area showed relatively limited differences across conditions. This suggests that the spatial organization of adhesions, rather than adhesion abundance alone, may be associated with the greater elongation observed on Nano. Thus, surface architectures sufficient to establish cellular orientation do not necessarily produce the same degree of change in cell morphology. Because aligned Schwann cells form Bands of Büngner that support regenerating axons following peripheral nerve injury, these results indicate that different topographical regimes may influence distinct aspects of Schwann cell organization relevant to regeneration.

Another notable finding was that neuronal responses to topographical cues were detectable before extensive neurite differentiation. After only 24 h of culture, the Submicro and Micro conditions showed greater cellular alignment, whereas process extension showed more modest differences across the topographical regimes, with the Submicro condition showing the greatest early process extension. These observations suggest that neuronal organization in response to anisotropic surface architecture is evident early, before more pronounced differences in neurite guidance emerge following differentiation. Such early cellular organization may influence subsequent neurite guidance and highlights the importance of considering multiple stages of neuronal development when evaluating biomaterial interfaces.

Following neuronal differentiation, differences across topographical regimes became more pronounced. Compared with the Planar and Nano groups, both the Submicro and Micro topographies showed greater directional persistence and lateral confinement. However, the Submicro group showed the greatest neurite extension while exhibiting guidance metrics comparable to those of the Micro group. These findings suggest that stronger confinement does not necessarily correspond to greater neurite outgrowth and that different topographical regimes may be associated with distinct patterns of neurite extension and directional guidance. Together with the Schwann cell results, these observations indicate that no single topographical regime produced the strongest response across all regenerative outcomes evaluated in this study.

The vinculin findings provide additional insight into how adhesion organization may contribute to distinct cellular responses to anisotropic topographies. Focal adhesions mechanically couple integrin-based cell–substrate interactions to the actin cytoskeleton and are therefore thought to play an important role in contact guidance on anisotropic surfaces [50, 51]. The distinct vinculin organization observed in neural-like cells is also consistent with the more dynamic and heterogeneous adhesion structures associated with neuronal processes and growth cones [52, 53]. Thus, Schwann and neuronal cells may engage the same anisotropic surface through different adhesion architectures, which could contribute to their distinct responses across the topographical regimes.

Beyond the biological findings, the fabrication strategy provides several practical advantages for biomaterial design. Unlike many conventional patterning techniques, the wrinkle-based approach enables predictable tuning of topographical dimensions simply by varying multilayer thickness while maintaining a common material composition and surface chemistry. Because wrinkle formation is driven by mechanical buckling rather than lithographic patterning, the approach is scalable and adaptable to substrates with diverse geometries. These characteristics make the platform well suited for systematically investigating topography-mediated cell responses and provide a foundation for future translation to more complex three-dimensional regenerative biomaterials.

This study has several limitations. First, RSC96 and SH-SY5Y cells are established cell lines and do not fully reproduce the phenotypes of primary Schwann cells and neurons. Future studies should validate these responses using primary or stem cell-derived neural cells. Second, neuronal and glial responses were evaluated independently to establish the responses of each cell type to the underlying topography, whereas peripheral nerve regeneration involves dynamic interactions between Schwann cells and regenerating axons. Future sequential co-culture studies could first establish aligned Schwann cell populations on the patterned substrates and then introduce neuronal cells, allowing neurite orientation to be evaluated relative to both Schwann cell organization and the underlying wrinkle axis. Such studies could determine whether Schwann cell-mediated guidance reinforces, modifies, or competes with direct topographical guidance. Third, the geometric parameters of the wrinkled surfaces are not independently controlled; changes in characteristic wrinkle wavelength are coupled with changes in ridge width, amplitude, and curvature. The observed cellular responses should therefore be interpreted across different topographical regimes rather than as effects of a single geometric parameter. Future fabrication strategies that provide greater independent control of these features will help determine their individual contributions. Fourth, the current work was performed on 2D substrates. Future studies should evaluate whether these topographical response trends translate to 3D nerve guidance conduits and other more physiologically relevant regenerative environments. Finally, although our findings suggest that focal adhesion organization may contribute to cellular responses to topography, the underlying molecular mechanisms remain to be determined.

In summary, this study demonstrates that engineered wrinkled topographies can provide physical guidance cues for both neuronal and glial cells. Rather than identifying a single optimal surface architecture, our findings show that different regenerative processes exhibit distinct responses to topographical feature-size regimes. By comparing how wrinkle dimensions are associated with Schwann cell organization, early neuronal alignment, and neurite guidance within a common material platform, this work provides insight that may inform the design of neural guidance biomaterials and regenerative interfaces.

## Experimental Section

### Materials

Ti_3_AlC_2_ MAX phase powders (300 mesh, ≥ 99%) were obtained from Laizhou Kai Kai Ceramic Materials Co. Clear heat-shrinkable polystyrene (PS) films were purchased from Grafix. All other materials were purchased from either Sigma-Aldrich or Fisher Scientific. All reagents were used as received without further purification. Ultrapure deionized water (18.2 MΩ, Milli-Q) was used throughout the study. SH-SY5Y neuroblastoma cells and RSC96 Schwann cells were obtained from ATCC. All cell culture reagents were used as received.

### MXene Synthesis

Ti_3_C_2_T_x_ MXene nanosheets were synthesized through selective etching of aluminum from Ti_3_AlC_2_ MAX phase. Briefly, 7.5 g of LiF was dissolved in 40 mL of 9 M HCl under stirring followed by gradual addition of 1.0 g of Ti_3_AlC_x_powder. The etching reaction was allowed to proceed for 24 h at 35 °C. Following etching, the reaction mixture was repeatedly washed with deionized water through centrifugation cycles until the supernatant reached near-neutral pH. Delamination of multilayer MXene was achieved through 1 h of bath sonication in ice, followed by centrifugation at 3500 rpm for 30 min to remove unexfoliated particles. The resulting supernatant containing few-layer Ti_3_C_2_T_x_nanosheets was collected and stored at 4 °C prior to use.

### Layer-by-Layer (LbL) Film Fabrication

Prestressed PS substrates were cut into rectangular films and cleaned with ethanol and deionized (DI) water for 15 min each prior to deposition. Substrates were plasma-treated (Tergeo-Plus, PIE Scientific) immediately before multilayer assembly to improve surface wettability. Plasma-treated PS films were first immersed in poly(diallyldimethylammonium chloride) (PDAC) for 30 min to deposit a positively charged base layer. Wrinkled multilayer coatings were fabricated through alternating immersive deposition of negatively charged MXene nanosheets and positively charged gentamicin sulfate (GS) solutions. Substrates were immersed in GS solution for 5 min, rinsed with deionized water, immersed in MXene solution for 5 min, and rinsed again to remove loosely adsorbed material. This deposition cycle constituted one bilayer.

Films containing 1, 3, 6, 10, and 20 bilayers were fabricated for topographical characterization studies. For biological experiments, planar coatings (20-bilayer) as well as nano (1-bilayer), submicro (10-bilayer), and micro (20-bilayer) wrinkled topographies were selected for further analysis (Table S1).

### Fabrication of Anisotropic Wrinkled Topographies

To generate aligned anisotropic topographies, multilayer-coated PS substrates were mechanically constrained along one axis using a custom clamping setup and thermally shrunk at 130 °C for 1 min. During thermal shrinkage, unconstrained substrate contraction generated compressive stress within the stiff multilayer coating, producing aligned wrinkle architectures through buckling instability. Resulting wrinkle features formed perpendicular to the direction of unconstrained shrinkage. Planar controls consisted of unshrunk multilayer-coated substrates (20BL) containing identical surface chemistry without anisotropic wrinkle formation.

### Film Characterization

Multilayer growth behavior was characterized using UV–vis spectroscopy (Agilent BioTek Synergy H1). Absorbance measurements were collected at 780 nm to quantify MXene multilayer growth. Absorbance values were measured for films containing 1, 3, 6, 10, and 20 bilayers. Three positions on three independently fabricated films were analyzed for each condition.

Surface morphology and wrinkle organization were characterized using scanning electron microscopy (SEM) (Tescan Mira3). Samples were sputter coated with a thin conductive metal layer prior to imaging. SEM images were acquired at multiple magnifications to evaluate wrinkle alignment and surface morphology. Wrinkle wavelength was quantified as the peak-to-peak distance between adjacent wrinkle ridges using a custom Python image-analysis script. Mean wrinkle wavelength was calculated from at least 100 individual peak-to-peak measurements across three independently fabricated films per condition. Distributions of the individual measurements were used to characterize local feature-size variation.

Mechanical properties were characterized by measuring the apparent Derjaguin–Muller–Toporov (DMT) modulus using AFM (Veeco Dimension Icon) in PeakForce Quantitative Nanomechanics (QNM) mode in air.

### RSC96 Schwann Cell Culture

RSC96 Schwann cells were cultured in Dulbecco’s Modified Eagle Medium (DMEM) supplemented with 10% fetal bovine serum (FBS) and 1% penicillin–streptomycin (Pen/Strep) under standard humidified culture conditions at 37 °C and 5% CO_2_. Cells were maintained in T75 tissue culture flasks and passaged using trypsin–EDTA upon reaching ~ 80–90% confluency. Cells were seeded directly onto planar and wrinkled substrates in complete growth medium for 48 h prior to imaging and analysis.

### SH-SY5Y Cell Culture and Differentiation

SH-SY5Y neuroblastoma cells were cultured in a 1:1 mixture of Dulbecco’s Modified Eagle Medium and Ham’s F12 nutrient mixture (DMEM/F12) supplemented with 10% FBS and 1% Pen/Strep. Cells were maintained under standard humidified culture conditions at 37 °C and 5% CO_2_ and passaged using trypsin–EDTA prior to reaching full confluency.

Prior to cell seeding, substrates were rinsed with sterile PBS and coated with extracellular matrix proteins (25 μg/mL collagen and 25 μg/mL laminin) to improve cellular attachment. For early morphology studies, SH-SY5Y cells were seeded onto planar and wrinkled substrates and cultured for 24 h in complete growth medium prior to imaging and orientation analysis. For neurite differentiation studies, SH-SY5Y cells were differentiated using a three-stage differentiation protocol. Cells were first seeded onto matrix-coated substrates for 24 h for initial adhesion. After 24 h, the complete media was aspirated and replaced with low serum (1% BSA) media containing 10 μM retinoic acid (RA) for 4 days, with an intermediate media change after 48 h. This procedure was followed by supplementation with brain-derived neurotrophic factor (BDNF) at 50 ng/mL in low serum media for an additional 48 h. At this point, the SH-SY5Y cells were defined as fully differentiated for subsequent processing and analysis.

### Immunofluorescence Staining

Cells were fixed with 4% paraformaldehyde for 15 min and permeabilized using 0.3% Triton X-100. For F-actin staining alone (RSC96 and early SH-SY5Y experiments), Alexa Fluor 647-conjugated phalloidin (ThermoFisher Scientific) (1:400) in 1% bovine serum albumin (BSA) was added to the cells for 30 min at RT, protected from light. For F-actin + beta3-tubulin staining (in differentiated SH-SY5Y cells), cells were first blocked with 5% goat serum for 1 h to block nonspecific binding sites. A mixture of anti-beta3-tubulin (TU-20) (Mouse IgG1 Monoclonal Antibody) (1:200) and Alexa Fluor 647-conjugated phalloidin (1:400) in 1% BSA and 0.3% Triton X-100 was then added to the cells and incubated overnight at 4 °C. The cells were then incubated with Alexa Fluor 555-conjugated goat anti-mouse IgG (1:500) for 1 h at RT. Samples were mounted using Prolong Gold Antifade with DAPI, and imaged using a fluorescence microscope (Axio Observer, Carl Zeiss Inc.). All staining steps were performed with minimal light exposure and with thorough PBS washes between all incubation steps.

For vinculin-positive adhesion analysis, cells were first fixed with ice-cold methanol for 15 min. Cells were then permeabilized and blocked with 5% goat serum and 0.3% Triton X-100 for 1 h. Anti-Vinculin (EPR8185) (Rabbit IgG Monoclonal Antibody) (1:250) and Alexa Fluor 647-conjugated phalloidin (1:400) in 1% BSA and 0.3% Triton X-100 was then added to the cells and incubated overnight at 4 °C. The cells were then incubated with Alexa Fluor 555-conjugated goat anti-rabbit IgG (1:500) for 1 h at RT. Samples were mounted using Prolong Gold Antifade with DAPI, and imaged using a fluorescence microscope (Axio Observer, Carl Zeiss Inc.). All staining steps were performed with minimal light exposure and with thorough PBS washes between all incubation steps.

### Cellular SEM Sample Preparation

For SEM analysis, samples were gently washed with phosphate-buffered saline (PBS) and fixed with 2.5% glutaraldehyde in PBS for 30 min at room temperature. Following fixation, samples were rinsed with PBS and treated with 1% osmium tetroxide in PBS for 30 min at room temperature. Samples were rinsed in PBS and then gradually dehydrated in a graded ethanol/ultrapure water series (30%, 50%, 70%, and 100%) for 5 min in each solution. Samples were then dried using a critical point dryer (Leica EM CPD300) and sputter coated with a thin layer of conductive metal prior to SEM imaging.

### Quantification of Cellular Alignment and Morphology

Cell orientation and morphology were quantified using ImageJ and CellProfiler. For anisotropic samples, cellular orientation was defined as the minor angle between the major axis of the cell nucleus and the wrinkle axis. For planar samples, cellular orientation was measured against the horizontal axis. Cells aligned within 15° of the wrinkle axis were classified as aligned. Schwann cell aspect ratio was calculated as the ratio between the major and minor axes of the fitted cellular ellipse. Rose plots were generated using the Matplotlib library in Python to visualize alignment distributions across experimental groups.

### Neurite Trajectory Analysis

Neurite trajectories were manually traced from fluorescence microscopy images (either F-actin or beta3-tubulin) using ImageJ. Total neurite length was defined as the full traced neurite path length, while straight-line distance was defined as the direct distance between neurite origin and endpoint. Neurite persistence ratio was calculated as the ratio between straight-line distance and total neurite path length. Absolute lateral deviation was calculated relative to the primary wrinkle axis to quantify neurite confinement and transverse trajectory wandering.

### Statistical Analysis

All quantitative data are presented as mean ± s.d. unless otherwise noted. Statistical analyses were performed using GraphPad Prism. Comparisons between multiple experimental groups were performed using one-way or two-way ANOVA, as appropriate, with post hoc multiple-comparison testing. Statistical significance was defined as *p* < 0.05.

## Supplementary Material

SI

The online version contains supplementary material available at https://doi.org/10.1007/s12195-026-00953-7.

## Figures and Tables

**Fig. 1:**
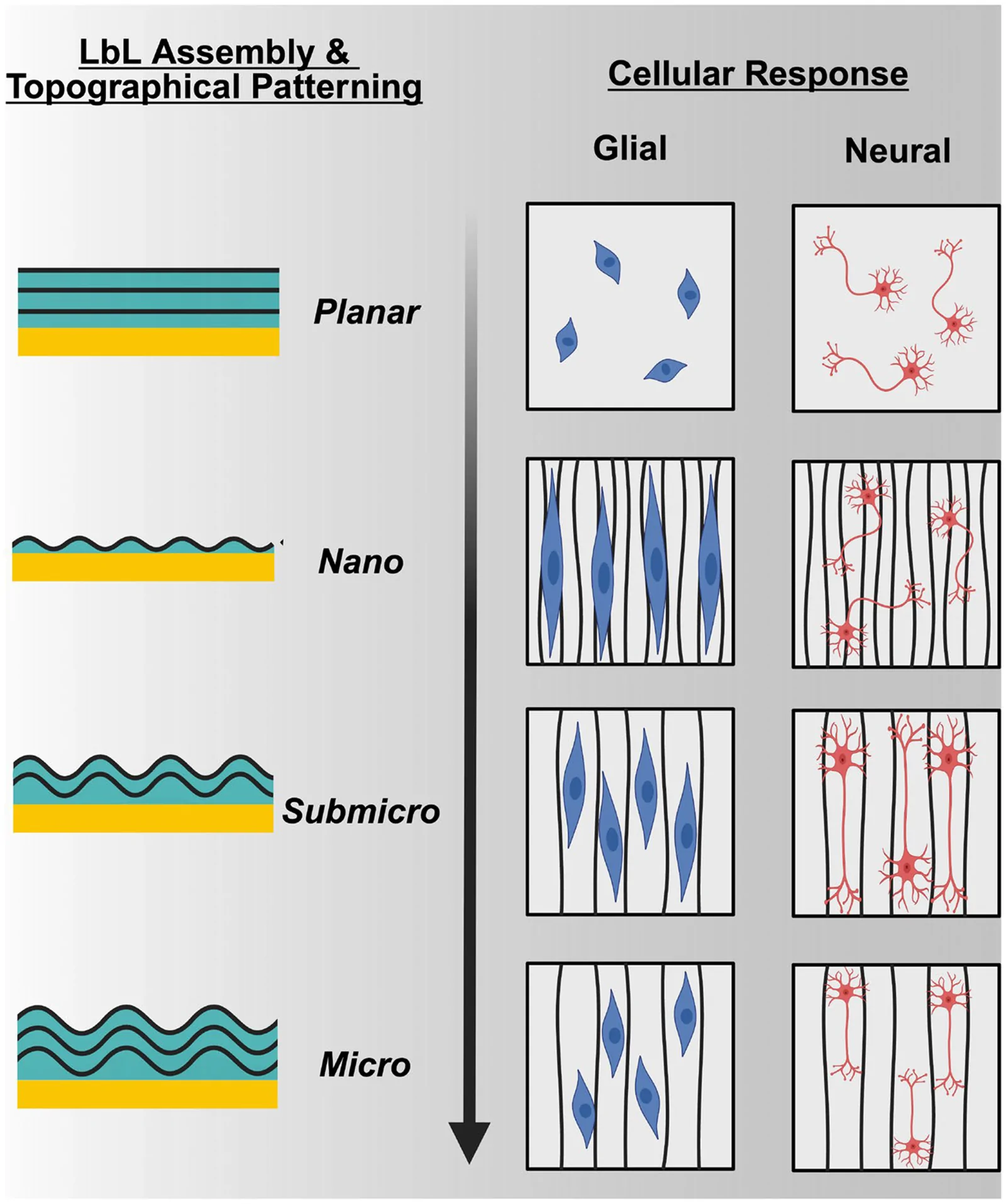
Conceptual framework of topography-mediated neuronal and glial organization. We hypothesize that anisotropic wrinkled topographies spanning distinct feature-size regimes differentially influence Schwann cell and neuron-like cell behavior through contact-guidance mechanisms. Systematic comparison across these topographical regimes enables evaluation of differences in cellular alignment, elongation, neurite extension, and directional guidance, providing insight into structure–response relationships relevant to neural guidance interfaces

**Fig. 2:**
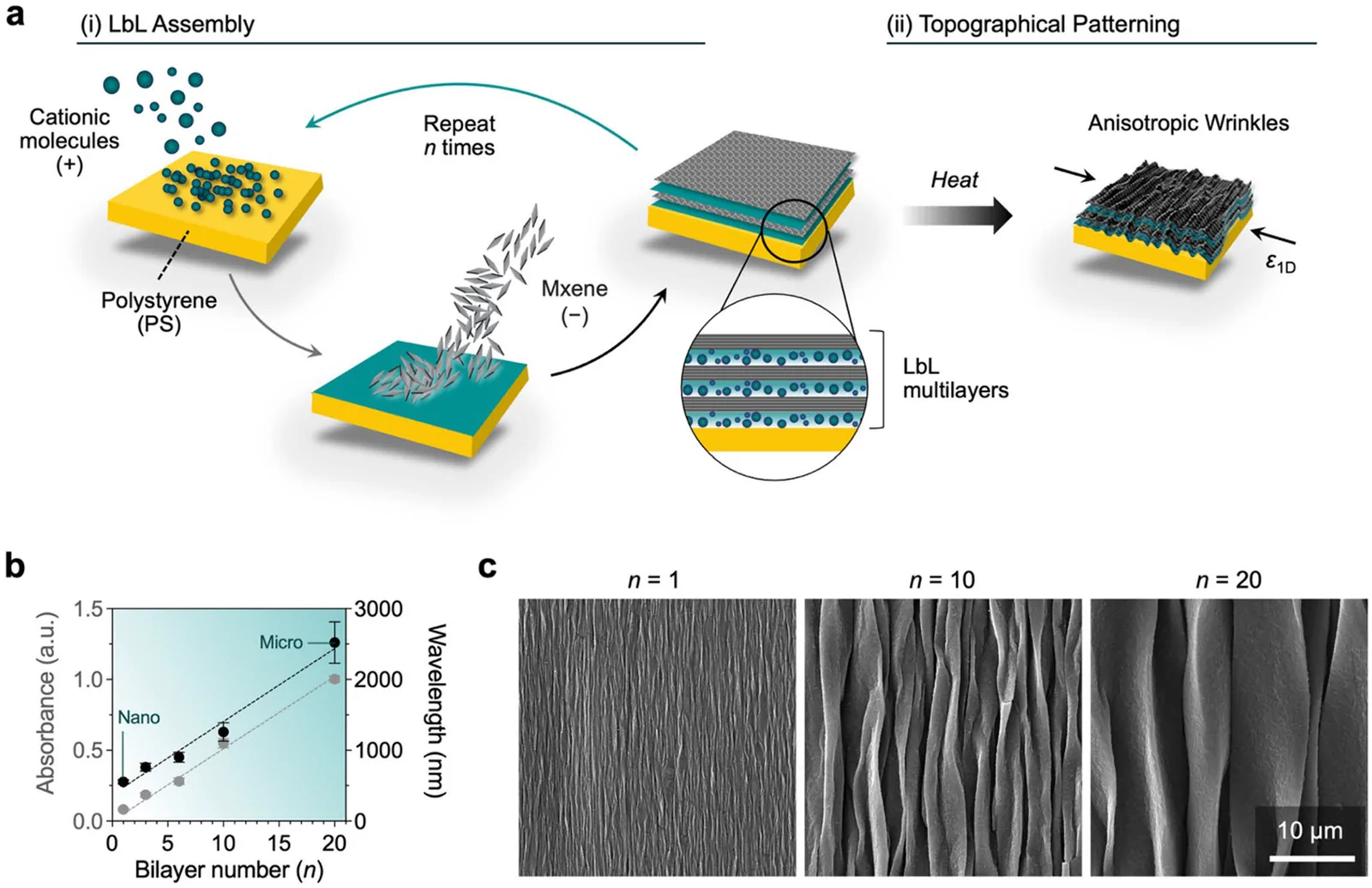
System fabrication and characterization of anisotropic wrinkled MXene multilayers. **a** Schematic of anisotropic wrinkled multilayer formation on polystyrene (PS) substrates. (i) LbL assembly showing controlled multilayer growth with increasing bilayer number and (ii) thermally induced topographical patterning under uniaxial strain (*ε*_1D_), resulting in aligned wrinkle architectures. **b** Absorbance at 780 nm of as-prepared multilayers (grey) and wrinkle wavelength (black) as a function of bilayer number. **c** Representative SEM images of wrinkled MXene multilayers. All experiments were performed in at least triplicate. Data are shown as mean ± s.d. a.u., arbitrary units

**Fig. 3:**
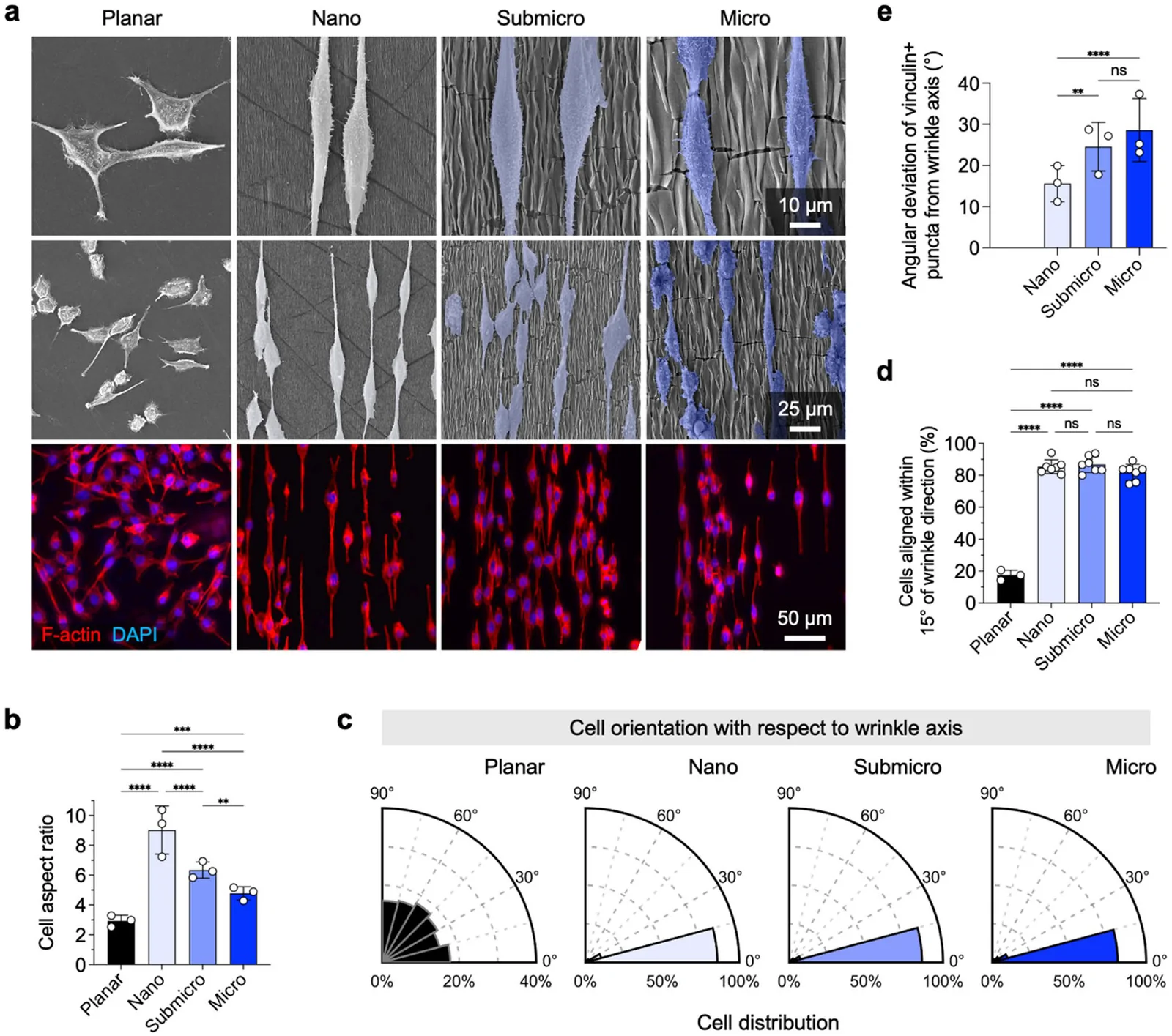
Feature-size-dependent Schwann cell responses to anisotropic wrinkled MXene topographies. **a** Representative SEM and IF images of RSC96 Schwann cells cultured for 48 h on Planar, Nano, Submicro, and Micro substrates. IF images show actin filaments (red) and nuclei (blue). **b** Quantitative assessment of cellular aspect ratio from IF images. **c** Rose plots depicting deviation of major cellular axis from wrinkle axis quantified from IF images. Cells parallel to the wrinkle axis are defined as 0°, while cells perpendicular to the wrinkle axis are defined as 90°. For the Planar condition, orientation was measured relative to the horizontal axis. **d** Percentage of cells aligned within 15° of the wrinkle axis (or horizontal axis for the Planar condition). **e** Angular deviation of Vinculin-positive puncta from the wrinkle axis; lower angular deviation indicates greater focal adhesion alignment with the wrinkle axis. Data are shown as mean ± s.d. from at least three biological replicates with at least 30 cells evaluated per replicate (****p < 0.0001; ***p < 0.001; **p < 0.01; *p < 0.05; ns, not significant)

**Fig. 4:**
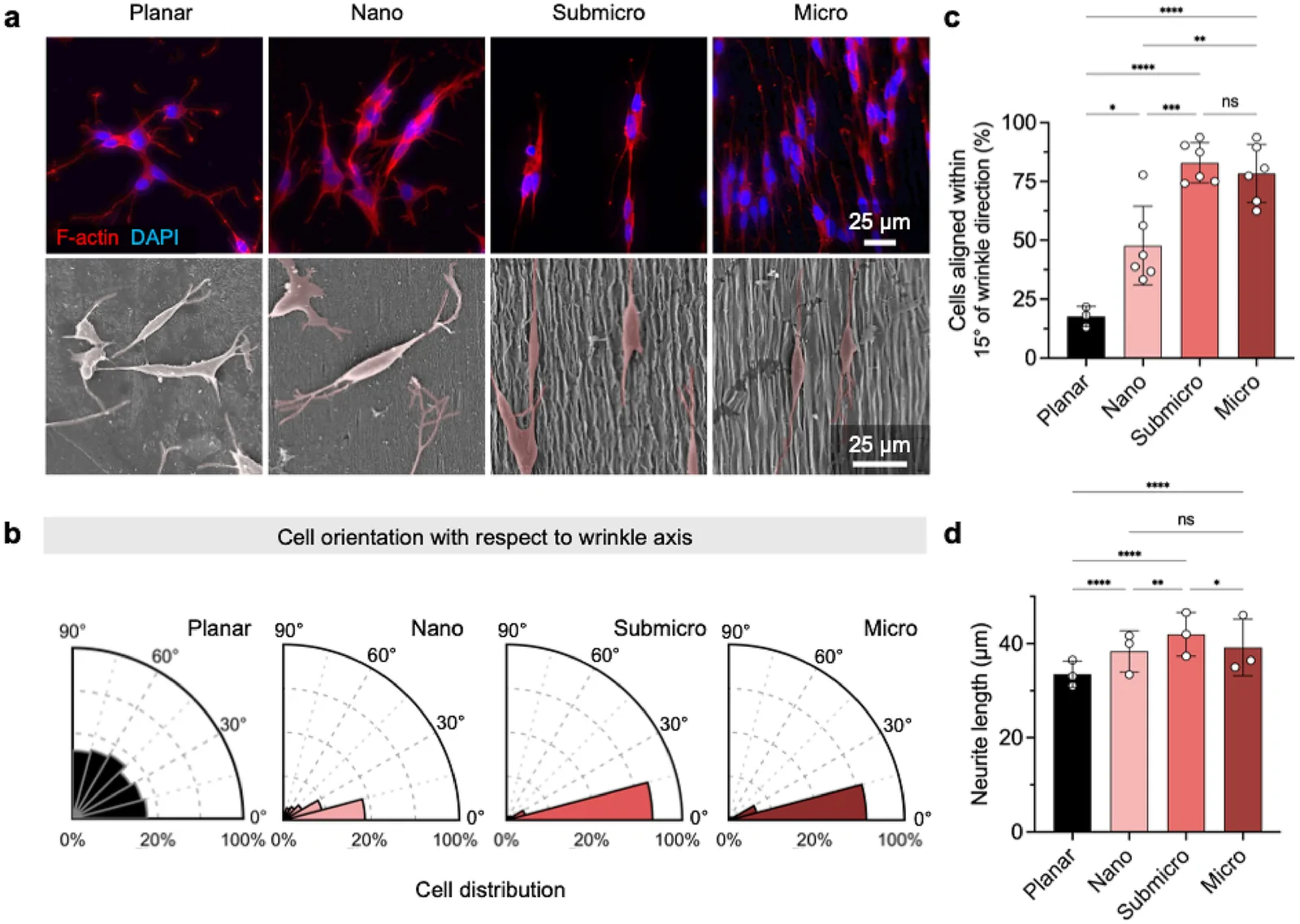
Feature-size-dependent early neuronal response to anisotropic wrinkled MXene topographies. **a** Representative IF and SEM images of SH-SY5Y cells cultured for 24 h on Planar, Nano, Submicro, and Micro substrates. IF images show actin filaments (red) and nuclei (blue). **b** Rose plots depicting deviation of major nuclear axis from wrinkle axis quantified from IF images. Cells parallel to the wrinkle axis are defined as 0°, while cells perpendicular to the wrinkle axis are defined as 90°. For the Planar condition, orientation was measured relative to the horizontal axis. **c** Percentage of cells aligned within 15° of the wrinkle axis (or horizontal axis for the Planar condition). **d** Quantitative analysis of neurite/process length from IF images. Data are shown as mean ± s.d. from at least three biological replicates with at least 30 cells/neurites evaluated per replicate (****p < 0.0001; ***p < 0.001; **p < 0.01; *p < 0.05; ns, not significant)

**Fig. 5:**
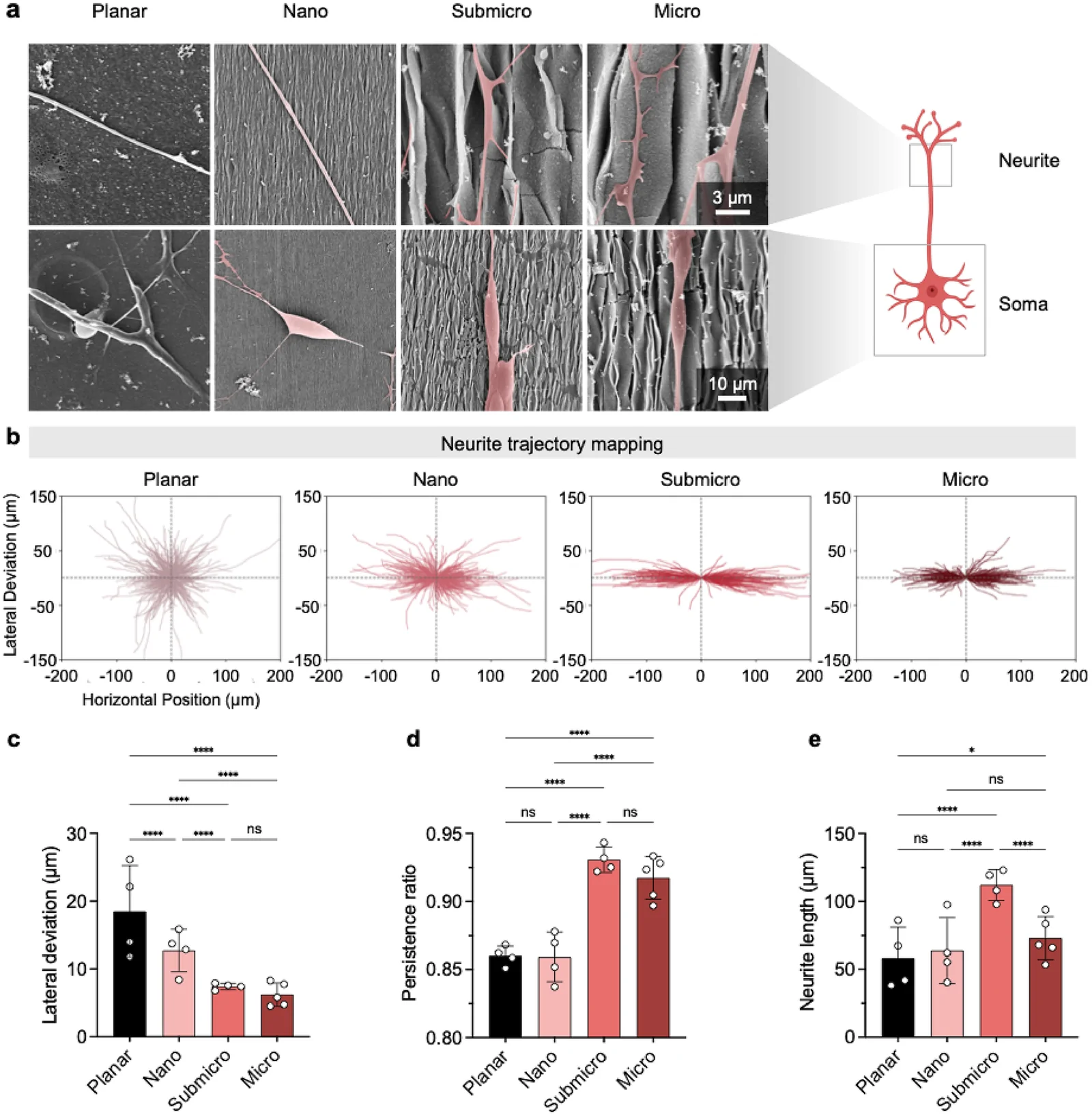
Feature-size-dependent regulation of neurite guidance and trajectory control. **a** Representative SEM images of differentiated SH-SY5Y cells cultured on Planar, Nano, Submicro, and Micro substrates. SEM panels are vertically split to highlight either cell neurites (top) or the cell soma (bottom). **b** Neurite trajectory maps showing neurite directional outgrowth relative to the wrinkle axis (or horizontal axis for Planar condition). **c** Quantification of neurite lateral deviation from the guidance axis. **d** Quantification of neurite persistence ratio. **e** Quantification of mean neurite length. Data are shown as mean ± s.d. from at least three biological replicates with at least 30 cells/neurites evaluated per replicate (****p < 0.0001; ***p < 0.001; **p < 0.01; *p < 0.05; ns, not significant)

## Data Availability

Relevant datasets from this study are available from the corresponding author upon reasonable request.

## References

[1] Lans J, Eberlin KR, Evans PJ, Mercer D, Greenberg JA, and Styron JF. A Systematic Review and Meta-Analysis of Nerve Gap Repair: Comparative Effectiveness of Allografts, Autografts, and Conduits. Plast. Reconstr. Surg. 151(5):814e, 2023. 10.1097/PRS.0000000000010088. 36728885

[2] Liao F; Zhou R; Chen S; Li X; Wu Y; Pang Q; Gu C; Zeng J; Yin S; Qiu S; Zhu Q; Zheng C. Peripheral Nerve Injury: Global, Regional, and National Incidence, Prevalence, and Disability from the Global Burden of Disease Study 2021 (1990–2021). Int. J. Surg. 10.1097/JS9.0000000000005217.

[3] Stocco E, Barbon S, Emmi A, Tiengo C, Macchi V, De Caro R, and Porzionato A. Bridging Gaps in Peripheral Nerves: From Current Strategies to Future Perspectives in Conduit Design. Int. J. Mol. Sci. 24(11):9170, 2023. 10.3390/ijms24119170. PMC10252835 37298122

[4] Chan YM, Judge NG, Hu Y, Willits RK, Li N, and Becker ML. Review of Gaps in the Clinical Indications and Use of Neural Conduits and Artificial Grafts for Nerve Repair and Reconstruction. Biomacromolecules. 26(7):3974–3997, 2025. 10.1021/acs.biomac.5c00558. PMC13107395 40562739

[5] Sanchez Rezza A; Kulahci Y; Gorantla VS; Zor F; Drzeniek NM. Implantable Biomaterials for Peripheral Nerve Regeneration–Technology Trends and Translational Tribulations. Front. Bioeng. Biotechnol. 2022, 10. 10.3389/fbioe.2022.863969. PMC9092979 35573254

[6] Carvalho CR; Oliveira JM; Reis RL. Modern Trends for Peripheral Nerve Repair and Regeneration: Beyond the Hollow Nerve Guidance Conduit. Front. Bioeng. Biotechnol. 2019, 7. 10.3389/fbioe.2019.00337. PMC6882937 31824934

[7] Hu X, Huang J, Ye Z, Xia L, Li M, Lv B, Shen X, and Luo Z. A Novel Scaffold with Longitudinally Oriented Microchannels Promotes Peripheral Nerve Regeneration. Tissue Eng. Part A. 15(11):3297–3308, 2009. 10.1089/ten.tea.2009.0017. 19382873

[8] Mobasseri A, Faroni A, Minogue BM, Downes S, Terenghi G, and Reid AJ. Polymer Scaffolds with Preferential Parallel Grooves Enhance Nerve Regeneration. Tissue Eng. Part A. 21(5–6):1152–1162, 2015. 10.1089/ten.tea.2014.0266. PMC4356253 25435096

[9] Xie J, MacEwan MR, Liu W, Jesuraj N, Li X, Hunter D, and Xia Y. Nerve Guidance Conduits Based on Double-Layered Scaffolds of Electrospun Nanofibers for Repairing the Peripheral Nervous System. ACS Appl. Mater. Interfaces. 6(12):9472–9480, 2014. 10.1021/am5018557. PMC4073935 24806389

[10] Huang C, Ouyang Y, Niu H, He N, Ke Q, Jin X, Li D, Fang J, Liu W, Fan C, and Lin T. Nerve Guidance Conduits from Aligned Nanofibers: Improvement of Nerve Regeneration through Longitudinal Nanogrooves on a Fiber Surface. ACS Appl. Mater. Interfaces. 7(13):7189–7196, 2015. 10.1021/am509227t. 25786058

[11] Miller C, Jeftinija S, and Mallapragada S. Micropatterned Schwann Cell-Seeded Biodegradable Polymer Substrates Significantly Enhance Neurite Alignment and Outgrowth. Tissue Eng. 7(6):705–715, 2001. 10.1089/107632701753337663. 11749728

[12] Chew SY, Mi R, Hoke A, and Leong KW. The Effect of the Alignment of Electrospun Fibrous Scaffolds on Schwann Cell Maturation. Biomaterials. 29(6):653–661, 2008. 10.1016/j.biomaterials.2007.10.025. PMC2713097 17983651

[13] Wang HB, Mullins ME, Cregg JM, McCarthy CW, and Gilbert RJ. Varying the Diameter of Aligned Electrospun Fibers Alters Neurite Outgrowth and Schwann Cell Migration. Acta Biomater. 6(8):2970–2978, 2010. 10.1016/j.actbio.2010.02.020. 20167292

[14] Hoffman-Kim D; Mitchel JA; Bellamkonda RV. Topography, Cell Response, and Nerve Regeneration. Annu. Rev. Biomed. Eng. 2010, 12 (Volume 12, 2010), 203–231. 10.1146/annurev-bioeng-070909-105351. PMC3016849 20438370

[15] Simitzi C, Ranella A, and Stratakis E. Controlling the Morphology and Outgrowth of Nerve and Neuroglial Cells: The Effect of Surface Topography. Acta Biomater. 51:21–52, 2017. 10.1016/j.actbio.2017.01.023. 28069509

[16] Liu F, Xu J, Wu L, Zheng T, Han Q, Liang Y, Zhang L, Li G, and Yang Y. The Influence of the Surface Topographical Cues of Biomaterials on Nerve Cells in Peripheral Nerve Regeneration: A Review. Stem Cells Int. 2021(1):8124444, 2021. 10.1155/2021/8124444. PMC8328695 34349803

[17] Corey JM, Lin DY, Mycek KB, Chen Q, Samuel S, Feldman EL, and Martin DC. Aligned Electrospun Nanofibers Specify the Direction of Dorsal Root Ganglia Neurite Growth. J. Biomed. Mater. Res. A. 83A(3):636–645, 2007. 10.1002/jbm.a.31285. 17508416

[18] Rajnicek A, Britland S, and McCaig C. Contact Guidance of CNS Neurites on Grooved Quartz: Influence of Groove Dimensions, Neuronal Age and Cell Type. J. Cell Sci. 110(Pt 23):2905–2913, 1997. 10.1242/jcs.110.23.2905. 9359873

[19] Tonazzini I; Cecchini A; Elgersma Y; Cecchini M. Interaction of SH-SY5Y Cells with Nanogratings During Neuronal Differentiation: Comparison with Primary Neurons. 10.1002/adhm.201300216. 24115396

[20] Walboomers XF, and Jansen JA. Cell and Tissue Behavior on Micro-Grooved Surfaces. Odontology. 89(1):0002–0011, 2001. 10.1007/s10266-001-8178-z. 14530915

[21] Curtis ASG, and Wilkinson CDW. Reactions of Cells to Topography. J. Biomater. Sci. Polym. Ed. 9(12):1313–1329, 1998. 10.1163/156856298X00415. 9860172

[22] Hsu S, Chen C-Y, Lu PS, Lai C-S, and Chen C-J. Oriented Schwann Cell Growth on Microgrooved Surfaces. Biotechnol. Bioeng. 92(5):579–588, 2005. 10.1002/bit.20634. 16261633

[23] Clark P, Connolly P, Curtis ASG, Dow JAT, and Wilkinson CDW. Cell Guidance by Ultrafine Topography in Vitro. J. Cell Sci. 99(1):73–77, 1991. 10.1242/jcs.99.1.73. 1757503

[24] Miller C, Jeftinija S, and Mallapragada S. Synergistic Effects of Physical and Chemical Guidance Cues on Neurite Alignment and Outgrowth on Biodegradable Polymer Substrates. Tissue Eng. 8(3):367–378, 2002. 10.1089/107632702760184646. 12167224

[25] Clark P, Connolly P, Curtis ASG, Dow JAT, and Wilkinson CDW. Topographical Control of Cell Behaviour: I. Simple Step Cues. Development. 99(3):439–448, 1987. 10.1242/dev.99.3.439. 3653011

[26] Clark P, Connolly P, Curtis AS, Dow JA, and Wilkinson CD. Topographical Control of Cell Behaviour: II. Multiple Grooved Substrata. Development. 108(4):635–644, 1990. 10.1242/dev.108.4.635. 2387239

[27] Asadi Tokmedash M, Kim C, Chavda AP, Li A, Robins J, and Min J. Engineering Multifunctional Surface Topography to Regulate Multiple Biological Responses. Biomaterials. 319:123136, 2025. 10.1016/j.biomaterials.2025.123136. PMC11893264 39978049

[28] Asadi Tokmedash M, Gillen O, and Min J. Scalable and Tunable 1D/2D Wrinkled MXene Topographies Regulate Osteoimmune Cell Interactions. ACS Appl. Bio Mater. 2026. 10.1021/acsabm.5c02475. PMC13720866 42253185

[29] Chavda A, Zhou X, Tokmedash MA, Moon JJ, and Min J. Tunable Wrinkled Topographies Direct Dendritic Cell Maturation and Immune Phenotypes. Acta Biomater. 212:428–443, 2026. 10.1016/j.actbio.2025.12.050. PMC12997553 41478520

[30] Guo R, Xiao M, Zhao W, Zhou S, Hu Y, Liao M, Wang S, Yang X, Chai R, and Tang M. 2D Ti3C2TxMXene Couples Electrical Stimulation to Promote Proliferation and Neural Differentiation of Neural Stem Cells. Acta Biomater. 139:105–117, 2022. 10.1016/j.actbio.2020.12.035. 33348061

[31] Nan L-P; Lin Z; Wang F; Jin X-H; Fang J-Q; Xu B; Liu S-H; Zhang F; Wu Z; Zhou Z-F; Chen F; Cao W-T; Wang J-G; Liu J-J. Ti3C2Tx MXene-Coated Electrospun PCL Conduits for Enhancing Neurite Regeneration and Angiogenesis. Front. Bioeng. Biotechnol. 2022, 10. 10.3389/fbioe.2022.850650. PMC8966647 35372318

[32] Cai J, Zhang H, Hu Y, Huang Z, Wang Y, Xia Y, Chen X, Guo J, Cheng H, Xia L, Lu W, Zhang C, Xie J, Wang H, and Chai R. GelMA-MXene Hydrogel Nerve Conduits with Microgrooves for Spinal Cord Injury Repair. J. Nanobiotechnology. 20(1):460, 2022. 10.1186/s12951-022-01669-2. PMC9617371 36307790

[33] Wang Z, Zheng Y, Qiao L, Ma Y, Zeng H, Liang J, Ye Q, Shen K, Liu B, Sun L, and Fan Z. 4D-Printed MXene-Based Artificial Nerve Guidance Conduit for Enhanced Regeneration of Peripheral Nerve Injuries. Adv. Healthc. Mater. 13(23):2401093, 2024. 10.1002/adhm.202401093. 38805724

[34] Liao M, Cui Q, Hu Y, Xing J, Wu D, Zheng S, Zhao Y, Yu Y, Sun J, and Chai R. Recent Advances in the Application of MXenes for Neural Tissue Engineering and Regeneration. Neural Regen. Res. 19(2):258, 2024. 10.4103/1673-5374.379037. PMC10503607 37488875

[35] Poudel I, Lee JS, Tan L, and Lim JY. Micropatterning-Retinoic Acid Co-Control of Neuronal Cell Morphology and Neurite Outgrowth. Acta Biomater. 9(1):4592–4598, 2013. 10.1016/j.actbio.2012.08.039. 22939924

[36] Encinas M, Iglesias M, Liu Y, Wang H, Muhaisen A, Ceña V, Gallego C, and Comella JX. Sequential Treatment of SH-SY5Y Cells with Retinoic Acid and Brain-Derived Neurotrophic Factor Gives Rise to Fully Differentiated, Neurotrophic Factor-Dependent. Human Neuron-Like Cells. J. Neurochem. 75(3):991–1003, 2000. 10.1046/j.1471-4159.2000.0750991.x. 10936180

[37] Targett IL, Crompton LA, Conway ME, and Craig TJ. Differentiation of SH-SY5Y Neuroblastoma Cells Using Retinoic Acid and BDNF: A Model for Neuronal and Synaptic Differentiation in Neurodegeneration. Vitro Cell. Dev. Biol. - Anim. 60(9):1058–1067, 2024. 10.1007/s11626-024-00948-6. PMC11534981 39017752

[38] Nissan I, Schori H, Bhooshan Kumar V, Antman Passig M, Shefi O, and Gedanken A. Topographical Impact of Silver Nanolines on the Morphology of Neuronal SH-SY5Y Cells. J. Mater. Chem. B. 5(47):9346–9353, 2017. 10.1039/C7TB02492D. 32264537

[39] Wu Z-Z, Zhao Y, and Kisaalita WS. Interfacing SH-SY5Y Human Neuroblastoma Cells with SU-8 Microstructures. Colloids Surf. B Biointerfaces. 52(1):14–21, 2006. 10.1016/j.colsurfb.2006.06.001. 16837176

[40] Litowczenko J, Maciejewska BM, Wychowaniec JK, Kościński M, Jurga S, and Warowicka A. Groove-Patterned Surfaces Induce Morphological Changes in Cells of Neuronal Origin. J. Biomed. Mater. Res. A. 107(10):2244–2256, 2019. 10.1002/jbm.a.36733. 31136088

[41] Buttiglione M, Vitiello F, Sardella E, Petrone L, Nardulli M, Favia P, d’Agostino R, and Gristina R. Behaviour of SH-SY5Y Neuroblastoma Cell Line Grown in Different Media and on Different Chemically Modified Substrates. Biomaterials. 28(19):2932–2945, 2007. 10.1016/j.biomaterials.2007.02.022. 17391751

[42] Sun Y, Zhang Y, Guo Y, He D, Xu W, Fang W, Zhang C, Zuo Y, and Zhang Z. Electrical Aligned Polyurethane Nerve Guidance Conduit Modulates Macrophage Polarization and Facilitates Immunoregulatory Peripheral Nerve Regeneration. J. Nanobiotechnology. 22(1):244, 2024. 10.1186/s12951-024-02507-3. PMC11089704 38735969

[43] Huang W-C, Yao C-K, Liao J-D, Lin C-CK, and Ju M-S. Enhanced Schwann Cell Adhesion and Elongation on a Topographically and Chemically Modified Poly(L-Lactic Acid) Film Surface. J. Biomed. Mater. Res. A. 99A(2):158–165, 2011. 10.1002/jbm.a.33090. 21976440

[44] Scaccini L, Mezzena R, De Masi A, Gagliardi M, Gambarotta G, Cecchini M, and Tonazzini I. Chitosan Micro-Grooved Membranes with Increased Asymmetry for the Improvement of the Schwann Cell Response in Nerve Regeneration. Int. J. Mol. Sci. 22(15):7901, 2021. 10.3390/ijms22157901. PMC8348329 34360664

[45] Jiang Z, Zhang Y, Wang Y, Wang S, Chang J, Liu W, and Han B. Multichannel Nerve Conduit Based on Chitosan Derivates for Peripheral Nerve Regeneration and Schwann Cell Survival. Carbohydr. Polym. 301:120327, 2023. 10.1016/j.carbpol.2022.120327. 36446493

[46] Shen H, Shang Y, Jia M, Gao H, Wang L, Liu Y, Wang H, Gong Z, Jin N, and Li G. Wireless Modulation of Magneto-Responsive Schwann Cells and DRG via Integrating into Topological Electrospun Ovalbumin Scaffolds under External Magnetic Fields. Colloids Surf. Physicochem. Eng. Asp. 726:137826, 2025. 10.1016/j.colsurfa.2025.137826.

[47] Zheng T, Wu L, Sun S, Xu J, Han Q, Liu Y, Wu R, and Li G. Co-Culture of Schwann Cells and Endothelial Cells for Synergistically Regulating Dorsal Root Ganglion Behavior on Chitosan-Based Anisotropic Topology for Peripheral Nerve Regeneration. Burns Trauma. 10:tkac030, 2022. 10.1093/burnst/tkac030. PMC9444262 36071954

[48] Liu H, Lv P, Zhu Y, Wu H, Zhang K, Xu F, Zheng L, and Zhao J. Salidroside Promotes Peripheral Nerve Regeneration Based on Tissue Engineering Strategy Using Schwann Cells and PLGA: In Vitro and in Vivo. Sci. Rep. 7(1):39869, 2017. 10.1038/srep39869. PMC5213129 28054637

[49] Huang ZY, Hong W, and Suo Z. Nonlinear Analyses of Wrinkles in a Film Bonded to a Compliant Substrate. J. Mech. Phys. Solids. 53(9):2101–2118, 2005. 10.1016/j.jmps.2005.03.007.

[50] Kerstein PC; Nichol RH; Gomez TM. Mechanochemical Regulation of Growth Cone Motility. Front. Cell. Neurosci. 2015, 9. 10.3389/fncel.2015.00244. PMC4493769 26217175

[51] Geiger B, Spatz JP, and Bershadsky AD. Environmental Sensing through Focal Adhesions. Nat. Rev. Mol. Cell Biol. 10(1):21–33, 2009. 10.1038/nrm2593. 19197329

[52] Mandal P, Belapurkar V, Nair D, and Ramanan N. Vinculin-Mediated Axon Growth Requires Interaction with Actin but Not Talin in Mouse Neocortical Neurons. Cell. Mol. Life Sci. 78(15):5807–5826, 2021. 10.1007/s00018-021-03879-7. PMC11071915 34148098

[53] Sydor AM, Su AL, Wang FS, Xu A, and Jay DG. Talin and Vinculin Play Distinct Roles in Filopodial Motility in the Neuronal Growth Cone. J. Cell Biol. 134(5):1197–1207, 1996. 10.1083/jcb.134.5.1197. PMC2120982 8794861

