# Supplementary material for: Tunable Wrinkled Topographies Reveal Feature-Size-Dependent Regulation of Neuronal and Glial Spatial Organization
Source: Cell Mol Bioeng. Author manuscript; Available in PMC 2026 Nov 11. (PMC13750836; doi:10.1007/s12195-026-00953-7)
Supplement: Supplementary file 1 [file NIHMS2219318-supplement-SI.docx]

**Supporting Information**

**Tunable Wrinkled Topographies Reveal Feature-Size-Dependent Regulation of Neuronal and Glial Spatial Organization**

Owen Gillen^1^, Jouha Min^1,2,3,4,5,6*^

1. Department of Chemical Engineering, University of Michigan, Ann Arbor, MI, USA
2. Department of Biomedical Engineering, University of Michigan, Ann Arbor, MI, USA
3. Macromolecular Science and Engineering Program, University of Michigan, Ann Arbor, MI, USA
4. Rogel Cancer Center, University of Michigan, Ann Arbor, MI, USA
5. Biointerfaces Institute, University of Michigan, Ann Arbor, MI, USA
6. Weil Institute for Critical Care Research and Innovation, Ann Arbor, MI, USA

* Corresponding author: Jouha Min, PhD

Jouha Min, PhD

Department of Chemical Engineering

University of Michigan

Ann Arbor, MI, USA

734-647-4821

[jouhamin@umich.edu](mailto:jouhamin@umich.edu)

**Figure S1. Distribution of wrinkle wavelength across different bilayer conditions.** Individual wrinkle wavelength measurements are shown for 1-, 10-, and 20-bilayer films (n ≥ 100 measurements per condition) across three independently fabricated samples.

**Figure S2. Apparent mechanical properties of planar and wrinkled substrates.** Normalized apparent DMT modulus of polystyrene (PS) substrates and MXene LbL films (1-, 10-, and 20-bilayer) under planar vs. 1D wrinkled conditions. Values are normalized to planar PS. Data are shown as mean ± s.d. from three independently fabricated substrates per condition.

**Figure S3. Wide view-field images of Nano and Micro substrates illustrating consistent morphology.**

**Figure S4. Vinculin-positive focal adhesion organization in RSC96 Schwann cells across topographical regimes. (a)** Representative vinculin immunofluorescence images of RSC96 Schwann cells cultured for 48 h on different substrates. **(b)** Number of vinculin puncta per cell. **(c)** Mean area of vinculin puncta. **(d)** Aspect ratio of vinculin puncta. **(e)** Angular deviation of vinculin puncta from the wrinkle axis; lower angular deviation indicates greater alignment with the wrinkle axis. Each point represents the mean from one independent biological replicate. Data are shown as mean ± s.d. from three biological replicates (****p < 0.0001, ***p < 0.001, **p < 0.01, *p < 0.05; ns, not significant).

**Figure S5. Vinculin localization in SH-SY5Y neural-like cells across topographical regimes.** Representative vinculin immunofluorescence images of SH-SY5Y cells cultured for 24 h on different substrates.

**Figure S6.** **IF images of beta3-tubulin-stained differentiated SH-SY5Y cells** on Planar, Nano, Submicro, and Micro topographies. Larger wrinkle dimensions promoted increasingly organized neuronal networks aligned with the wrinkle direction (yellow arrows), consistent with the quantitative trajectory analyses shown in Figure 5. Scale bar = 100 μm.

**Table S1. Summary of experimental groups**

| **Group** | **LbL bilayers** | **Thermal Shrinkage** | **Surface  Topography** | **Mean wrinkle  Wavelength (nm)** |
| --- | --- | --- | --- | --- |
| Planar | 20 | No | Flat | N/A |
| Nano | 1 | Yes | 1D wrinkled | ~500 |
| Submicro | 10 | Yes | 1D wrinkled | ~1200 |
| Micro | 20 | Yes | 1D wrinkled | ~2500 |
